# Challenging the TAVR-Centric Approach in Asymptomatic Severe AS

**DOI:** 10.1016/j.jacadv.2025.102519

**Published:** 2026-01-19

**Authors:** David Rose, Grzegorz Laskawski, Cristiano Spadaccio

**Affiliations:** a Lancashire Cardiac Centre, Blackpool Victoria Hospital, Blackpool, United Kingdom; b University of Cincinnati, College of Medicine, Cincinnati, USA

We read with interest the article of Dayan et al,^1^ which provides a comprehensive review of early intervention compared with conservative management in asymptomatic severe aortic stenosis (AS). Although the authors appropriately highlight mortality benefits in the RECOVERY trial, we believe a critical perspective, particularly regarding procedural modality and patient selection, requires emphasis.

The RECOVERY trial demonstrated a 61% mortality reduction with early surgical aortic valve replacement (SAVR), the largest survival benefit reported to date. In contrast, EARLY TAVR showed no mortality difference despite early quality-of-life gains, which converged by 2 years.^2^ This divergence suggests that procedural modality significantly influences outcomes, yet the review does not sufficiently distinguish between transcatheter and surgical approaches in younger lower-risk patients.

In patients under 65 years, the median age 64 in RECOVERY, long-term durability and reintervention risk are paramount. Transcatheter valves carry higher rates of paravalvular leak, conduction disturbances, and their durability beyond 10 to 15 years remains to be established.^3^ SAVR, particularly with modern rapid-deployment valves, offers superior hemodynamics and longevity. Moreover, minimally invasive SAVR (mini-SAVR) via right anterior thoracotomy or upper hemisternotomy is now routine in high-volume centers, achieving short intensive care unit stays, rapid recovery, and excellent cosmesis.^4^ These advances mitigate traditional concerns about surgical trauma, yet are absent from the discussion.

Importantly, the historical surgical benchmark cited in current guidelines and comparative studies, often implicitly equated with median sternotomy, is outdated and never rigorously defined in the minimally invasive era.^5^ Without a contemporary and standardized surgical reference, TAVR comparisons risk being anchored to an obsolete procedural paradigm, skewing interpretation and heart team decision-making. Procedural access should be recognized as a modifiable variable, not a fixed limitation of surgery.

We further note the absence of patient-centered outcomes such as return-to-work rates or physical function, which are highly relevant in younger active individuals. Conservative management imposes psychological burden and surveillance fatigue, yet these are rarely quantified.

Future trials must stratify by access strategy and incorporate patient-reported outcomes. Shared decision-making tools should reflect modern surgical realities. Minimally invasive SAVR represents a compelling option, offering mortality benefit, durability, and acceptable recovery, particularly in patients where lifelong valve performance matters most.

We urge a recalibration of the early intervention paradigm: not simply *when* to intervene, but *how* and by whose standards the burden of procedure is judged. Establishing a contemporary surgical benchmark is essential to ensure fair, clinically meaningful comparisons as we refine early intervention strategies for asymptomatic AS.

## Declaration of Generative AI and AI-Assisted Technologies in the Writing Process

During the preparation of this work the author used ChatGPT, OpenAI, San Francisco, CA to assist with grammar, spelling, and stylistic editing of the manuscript. The author reviewed and edited the content as needed and takes full responsibility for the content of the published article.
